# The Cuts Selection Method Based on Histogram Segmentation and Impact on Discretization Algorithms

**DOI:** 10.3390/e24050675

**Published:** 2022-05-11

**Authors:** Visnja Ognjenovic, Vladimir Brtka, Jelena Stojanov, Eleonora Brtka, Ivana Berkovic

**Affiliations:** 1 Technical Faculty “Mihajlo Pupin” Zrenjanin, University of Novi Sad, 21102 Novi Sad, Serbia; visnjao@tfzr.uns.ac.rs (V.O.); vladimir.brtka@tfzr.rs (V.B.); jelena.stojanov@uns.ac.rs (J.S.); ivana.berkovic@tfzr.rs (I.B.); 2 Faculty of Traffic Engineering, University of East Sarajevo, 74000 Doboj, Bosnia and Herzegovina

**Keywords:** data discretization, groups of histograms, cuts, MD algorithm, entropy

## Abstract

The preprocessing of data is an important task in rough set theory as well as in Entropy. The discretization of data as part of the preprocessing of data is a very influential process. Is there a connection between the segmentation of the data histogram and data discretization? The authors propose a novel data segmentation technique based on a histogram with regard to the quality of a data discretization. The significance of a cut’s position has been researched on several groups of histograms. A data set reduct was observed with respect to the histogram type. Connections between the data histograms and cuts, reduct and the classification rules have been researched. The result is that the reduct attributes have a more irregular histogram than attributes out of the reduct. The following discretization algorithms were used: the entropy algorithm and the Maximal Discernibility algorithm developed in rough set theory. This article presents the Cuts Selection Method based on histogram segmentation, reduct of data and MD algorithm of discretization. An application on the selected database shows that the benefits of a selection of cuts relies on histogram segmentation. The results of the classification were compared with the results of the Naïve Bayes algorithm.

## 1. Introduction and Main Idea

Numerous machine learning methods can work only with the discrete attribute values. Therefore, the transformation of continuous attribute values into discrete ones, consisting of interval sets, is necessary prior to the machine learning process. This process, known as the data discretization, is essential in data preprocessing, not only because certain machine learning methods do not work with continuous attribute values but also because the data transformed into interval sets are cognitively relevant for human interpretation. The result of data discretization is a set of points that allocate data into intervals.

Having in mind the fact that the discretization process searches for partitions of the attribute domains and equalizes the values within each interval, the problem of discretization can be defined as a problem of searching for the relevant sets of cuts on the attribute domains. The quality of a discretization is often measured in relation to the result of classification.

There are some investigations on the influence of data distribution on the discretization algorithm results. The analysis is primarily an attempt to group data distribution by its significant features in relation to process of cut generation. Determining such important characteristics includes the experimental comparison of various distributions—histograms and cuts obtained through a concrete discretization algorithm.

Since 1999, a CRISP-DM [1] standard has been defined, clearly separating Data Understanding from Data Preparation. Data Understanding is the activity revealing the very essence of the data, while Data Preparation includes more activities that may cause data loss. The question is: Should the Data Understanding process be included in discretization algorithms?

In Section 2 is shown as the starting point of this research. Section 3 presents a histogram classification based on the number of unimodal parts. Section 4 indicates possible cuts locations on a histogram obtained by discretization’s algorithm. Section 5 emphasizes the main empirical research, while Section 6 shows an application of the new method. An application of the Cuts Selection Method on particular data sets is shown in Section 7. The results of the classification were compared with the results of the Naïve Bayes algorithm. Section 8 is Discussion and Conclusions.

## 2. Related Research

The papers presenting an analysis of the histogram impact on discretization algorithms will be briefly shown. 

**Figure 1:**
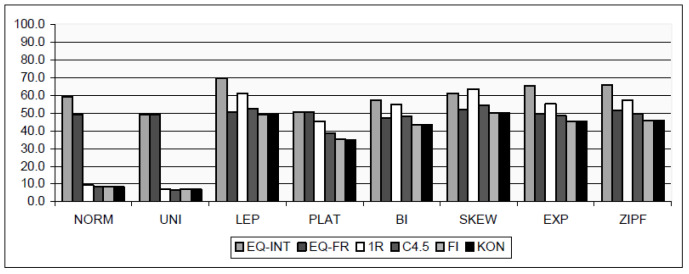
Average root relative squared error for all distributions and discretization methods, from [2].

In [2], the impact of concrete distributions to the six discretizations is shown. The data is synthesized such that their distributions are: Normal, Uniform, Leptokurtic, Platykurtic, Bimodal, Skewed, Exponential and Zipf. The used discretization algorithms are: Equal Interval Width, Equal Frequency, Holte’s 1R Discretizer, C4.5 Discretizer, Fayyad and Irani’s Entropy Based MDL Method and Kononenko’s Entropy Based MDL Method. By means of relative squared error and certain distribution, statistical parameters of classification quality are extracted; see Figure 1. Three of the six discretization algorithms are based on entropy (C4.5 Discretizer, Fayyad and Irani’s Entropy Based MDL Method and Kononenko’s Entropy Based MDL Method).

In rough set theory, within the Maximal Discernibility algorithm (MD algorithm), the relationship between data distribution and large databases using the median was investigated [3]. This is a statistical analysis of the distribution. The obtained result is related to the interval that contains the best cut, so histogram analysis is not necessary.

Data streams were investigated in [4], and an incremental algorithm was proposed. This algorithm slightly modifies data distribution so that discretization algorithm produces better results. In this case, a recursive entropy discretization algorithm was used.

**Figure 2:**
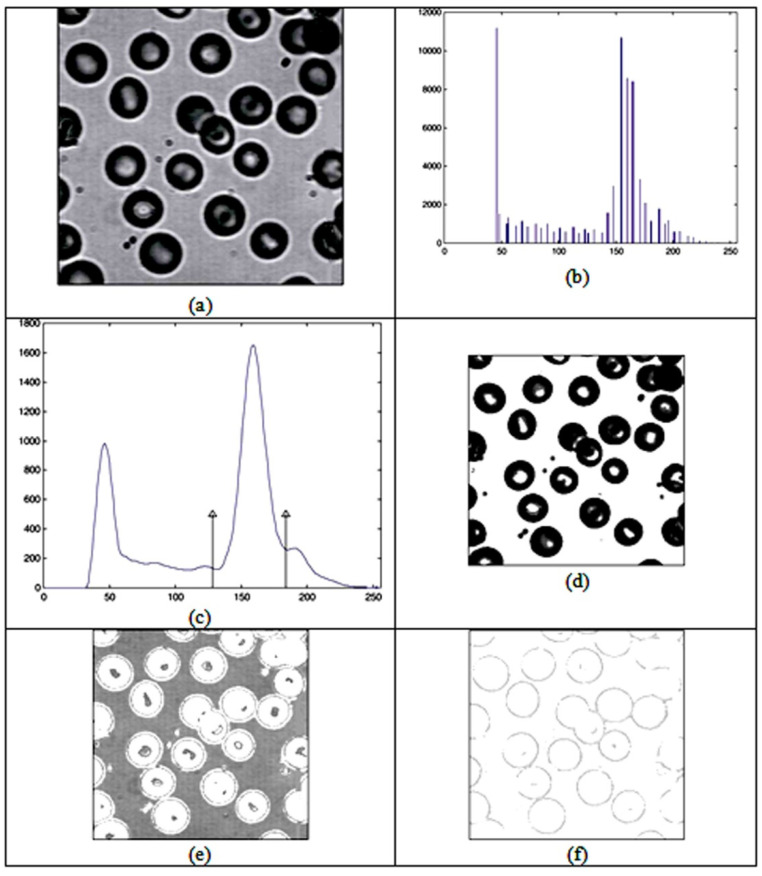
(**a**) Original image of blood; (**b**) gray-level histogram of the image; (**c**) smoothed histogram with threshold values; (**d**) the blood cells; (**e**) blood-plasma; and (**f**) the cell membrane (from [5]).

Investigations concerned with image segmentation based on the segmentation of the histogram had a major influence on this research. In [5], an analysis of the blood image was made with the histogram having two thresholds: Figure 2 shows a blood image (a), a histogram (b), a smooth curve obtained by a histogram (c) and images obtained on the basis of histogram segmentation. Based on the first part of the segmentation, a blood cell image (d) was obtained; based on the second part of the segmentation, a blood plasma image (e) was obtained; and based on the third part of the segmentation, an image of membrane cells (f) was obtained. The used sample has multimodal distribution with only three clusters, but it well reflects the multilevel thresholding technique presented in [5] where each cluster is analyzed by its deterministic parameters (mean, variance and probability). Based on the parameters, the thresholds were extracted.

If the segmentation of image histograms can extract clusters, as in Figure 2d–f, then what kind of connection exists between the segmentation of the data histogram and data clusters? The basic concept of this paper will be generalized to any data. Data discretization is closely related to histogram segmentation [6]. The sample from [5] is used only as a motivation for applying an analogous method on general-type data. Details of the multilevel threshold technic are not of the interest, for the method proposed in this paper differs significantly.

## 3. Histogram Classification

For a histogram obtained from real-life data, the estimation of similarity to some mathematical distribution is not productive. Namely, these histograms just partially correspond to some mathematical distribution and may, to some extent, be similar to more than one mathematical distribution.

**Figure 3:**
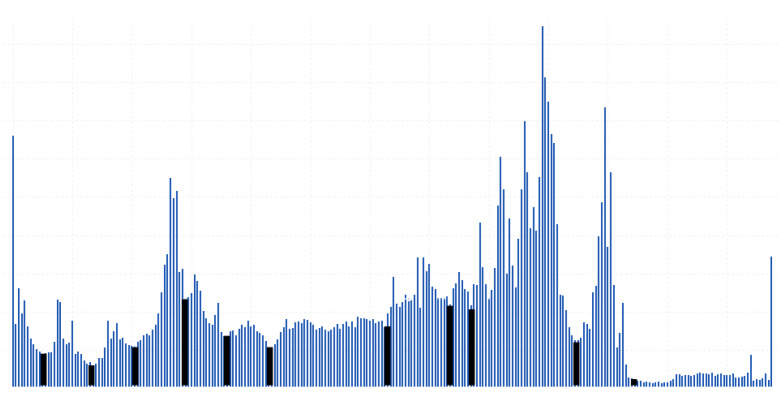
Histogram segmentation of real-life data.

In addition, histogram segmentation of real-life data may be similar to many mathematical distributions so that each segment is similar to a different distribution, as shown in Figure 3.

In [6], it was noticed that cuts generated by the MD algorithm are found after peaks. In the case of bimodal distribution, the cuts would probably be at a minimum between two maximums.

On the other side, investigations of image segmentations have confirmed that image segmentation thresholds are located in the valleys between histogram peaks. In addition to the peaks themselves, it is important to investigate the peaks surrounding. In [7], a thresholding technique is proposed. The technique is based on peak findings, the region initialization and the merging process. In image processing, segmentation is a central task for image analysis and pattern recognition. A popular tool for real-time image processing is a global histogram of a digital image [8]. Thus, histogram thresholding is a segmentation technique that looks for the peaks and valleys of a histogram [9]. The threshold selection of an image histogram deals with clustering [10,11], hierarchical clustering (image segmentation by histogram thresholding using hierarchical cluster analysis) [12], classical rough sets [13], rough sets combined with other techniques [14], and deep learning [15].

In the threshold selection analysis, unimodal and multimodal histograms are of interest for statistical and practical reasons [16]. Therefore, histograms in this research are classified as unimodal and multimodal. Another reason to group histograms as unimodal and multimodal is connected to cuts generated by the MD algorithm [6] and the equivalence of image segmentation (pixel segmentation) and data segmentation.

So, there are two groups of histograms and their corresponding distributions: the first and second types of histograms, as described in further lines.

### 3.1. First Type

**Figure 4:**
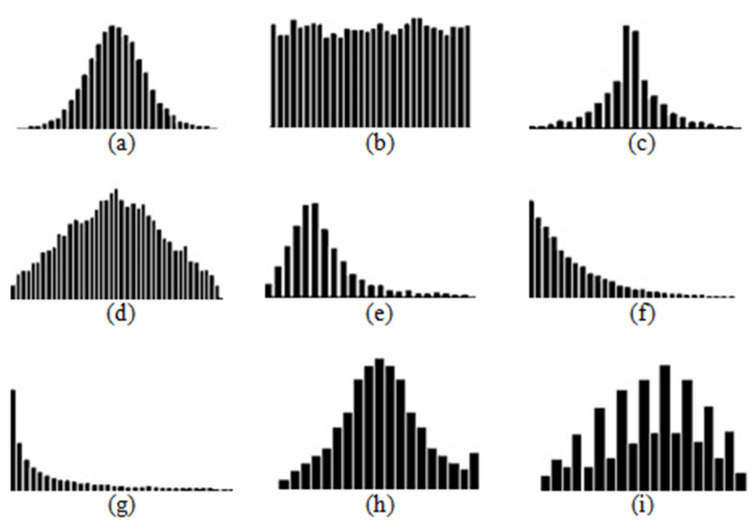
First type of histograms (normal (**a**), uniform (**b**), leptokurtic (**c**), platykurtic (**d**), skewed (**e**), exponential (**f**), zipf (**g**), edge peak (**h**) and comb (**i**) distributions).

First-type histograms are similar to the normal (Figure 4a), uniform (Figure 4b), leptokurtic (Figure 4c), platykurtic (Figure 4d), skewed (Figure 4e), exponential (Figure 4f), zipf (Figure 4g), edge peak (Figure 4h) and comb (Figure 4i) distributions. These distributions have no distinct peaks with minimums between them, although the comb distribution has indistinct peaks.

### 3.2. Second Type

**Figure 5:**
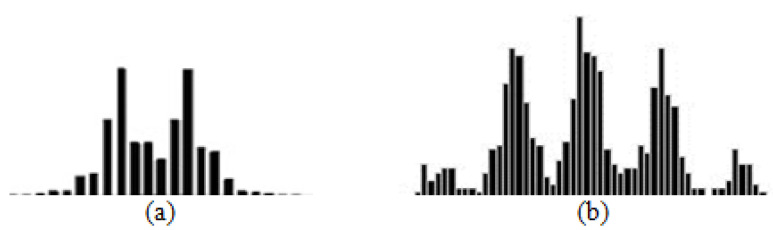
Second type of distributions (bimodal (**a**) and multimodal distributions (**b**)).

**Figure 6:**
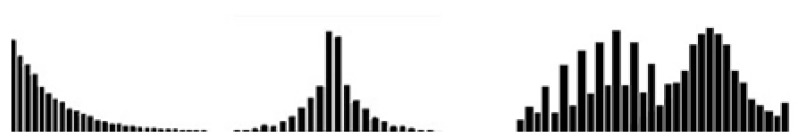
Examples of atypical multimodal distributions.

Second-type histograms are similar to bimodal (Figure 5a) or multimodal distributions (Figure 5b) with an arbitrary number of local maximums. In the case of multimodal distribution, any combination of the distributions from the first group, with or without a period of interruption, is possible as in Figure 6.

To investigate the position of the cuts in both types of histograms, ten data tables were used. All data tables consist of multiple condition attributes and one decision attribute. This is used to estimate the value of the decision attribute based on the values of the condition attributes. Four out of ten data tables have all or a majority of condition attributes whose distributions are of the first type. Additionally, four data tables have all or a majority of condition attributes whose distributions are of the second type. The two remaining data tables are chosen such that condition attributes evenly belong to both types.

Based on the experiment on the ten data tables, it will be determined how distributions of data affect the position of the cuts on histograms for a certain discretization algorithm.

## 4. Location of the Cuts on the Histogram

An analysis of the location of the cuts obtained using the MD algorithm and the algorithm based on the entropy on ten tables with different distributions was made. The used tables were obtained via UC Irvine Machine Learning Repository [17], as shown in Table 1. The basic characteristics of the data tables and the numbers of numeric attributes corresponding histogram type are listed in Table 1. EasyFit software [18] was used to generate histograms and to classify them by the types.

The data discretization was achieved by the following algorithms: the entropy algorithm [19] and MD algorithm developed in rough set theory [3]. For the discretization, the system Rosetta [20] was used.

The MD algorithm is a greedy algorithm for determining the minimum coverage set of objects from different classes of decision attributes. The MD algorithm creates initially a Boolean function from the set of candidate cuts and then computes a prime implicant of the function. Finally, the set of candidate cuts is defined by the Naive discretization algorithm [20]. MD algorithm combines the initial set of candidate cuts by using the Boolean reasoning procedure [3], which rejects certain cuts so that a small subgroup of cuts is formed. The obtained small subset is a minimal one that keeps the discernibility inherent in the data table.

The concept of entropy originates from information theory [21]. The entropy algorithm [19] is based on the recursive partitioning of the set of values of each attribute so that the local measure of entropy is optimal. The minimum length principle defines a criterion that stops the partition process.

### Histograms and Cuts—An Example

**Figure 7:**
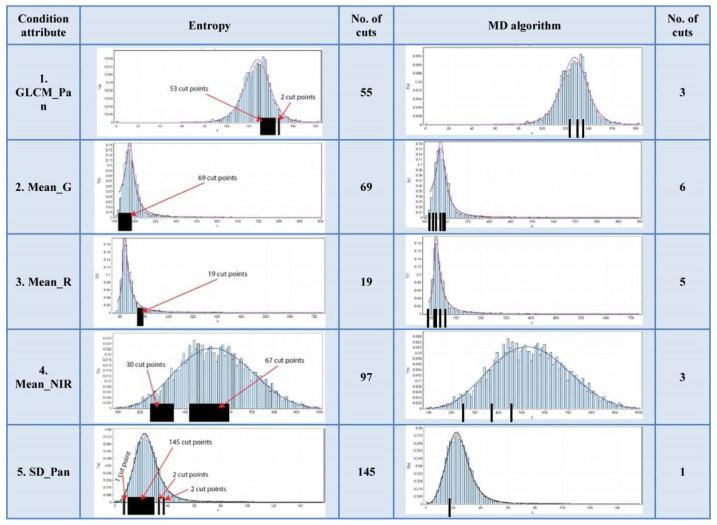
Cuts positioning on the histogram of the Wilt Data Set.

**Figure 8:**
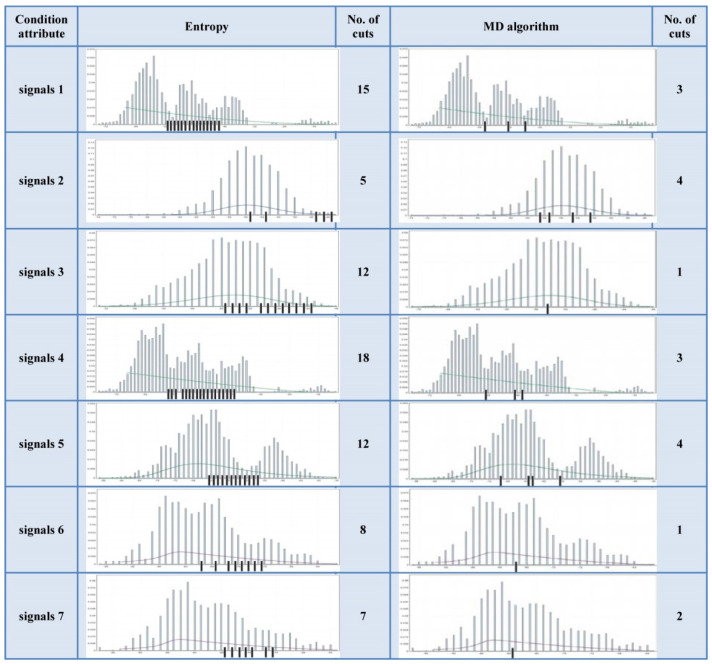
Cuts positioning on the histogram of the Wireless Indoor Localization Data Set.

**Figure 9:**
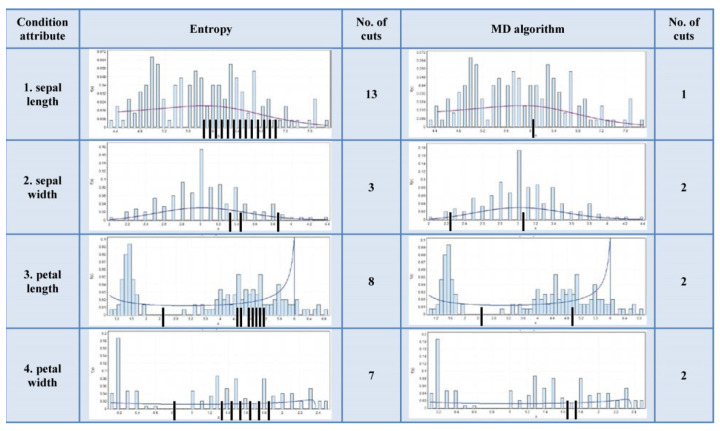
Cuts positioning on the histogram of the Iris Data Set.

The Wilt Data Set has all of attributes’ histograms from the first type. The Wireless Indoor Localization data table has a majority of the attributes’ histograms from the second type. The Iris data table has the attributes’ histograms evenly distributed to both types. These data tables histograms are shown in Figure 7, Figure 8 and Figure 9.

According to Figure 7, it can be noticed that the number of cuts of the MD algorithm is significantly lower than in the case of the entropy algorithm. According to Figure 8 and Figure 9, it is obvious that the number of cuts generated by the MD algorithm is in direct proportion to the segmentation of the multimodal distribution.

## 5. Influences—Research

According to Section 4, the cuts location on histograms, reduct results and classification results are closely related. This section explains the relation.

### 5.1. Cuts Location on Histograms

It is concluded that

- The cuts obtained by the entropy algorithm divide the histogram interval very irregularly, especially in the case of multimodal distributions (there is a big disproportion in the number of objects that determine the cuts);
- The cuts obtained by the MD algorithm are located after the local maximums or in thresholds of multimodal distribution.

A correlation in multimodal data is a topic of interest. The paper [22] confirms the advantages of base splitting by histogram segmentation with a significantly higher correlation on unimodal parts compared to the whole multimodal range. Additionally, this paper provides insight into the practical applications of data splitting, according to unimodal parts of histograms. Only on the data belonging to one unimodal part of the histogram can a significant correlation be seen.

### 5.2. Reduct Results

Condition attributes that describe the entire data table and generate the decision rules create a reduct [3]. The data table reduct derived by the application of the rough set theory was observed regarding to the type of data distribution. The results are given in Table 2. If only the reduct set attributes are considered, the following is noticed:

- In most cases, the reducts contain the attributes that correspond to second type of histogram;
- Compared to the MD algorithm, the entropy algorithm produces a reduct set with a larger number of attributes.

### 5.3. Classification Results Related to the Type of Histogram

The ten datasets were classified by the Rosetta system [20]. The classification procedure includes following:

- Datasets are split in two sets, the training set and the test set (50%:50%);
- The reduct is calculated for the training set by the variation of the Johnson’s reduct set calculation algorithm;
- IF THEN rules are obtained according to the previously calculated reduct set;
- The test set is classified by applying previously generated IF THEN rules.

Classification results are presented by a confusion matrix that describes the number of objects that are classified, as well as the number of unclassified objects. Therefore, it is possible to observe the number of objects that have been correctly and incorrectly classified (see Table 3). Additionally, the percentage of imprecise rules, those having an OR logical operator included in the THEN part of the rule, are considered. These rules are named inconsistent or imprecise [23], while the classification is accurate enough [3].

The analysis of the reduct sets, number of decision rules, the percentage of imprecise rules, and the results of the confusion matrices, for both of the observed algorithms, impose the following conclusions:

- The discretization of the dataset related to the second histogram type by use of the MD algorithm produces a larger reduct set, resulting in a larger number of rules and a worse classification;
- When discretizing a dataset of the second type by the entropy algorithm, more imprecise rules are obtained (compared to the MD algorithm), so the classification result is better.

### 5.4. Summarized Reserach

Based on the overall results, the importance of cuts that are simultaneously the thresholds of the multimodal distribution segmentation is emphasized. For the MD algorithm, it can be concluded that among all generated cuts, most of the thresholds of the multimodal distribution are retained, which is not the case with the entropy algorithm. In relation to the segmentation of the histogram of image that generates clusters, the segmentation of the histogram of non-image data allows for the allocation of data clusters. This is why the reduct set is made mostly by attributes with a multimodal distribution (from the second type). In the case where the reduct set is composed by the second-type attributes, with cuts corresponding to the histogram segmentation thresholds, a high precision of the classification rules can be expected, as shown in Table 3. This is especially confirmed with the MD algorithm.

In the case of selecting only those cuts that are simultaneously the thresholds of the multimodal distribution,

- The reduct set is minimal;
- The classification rules are sufficiently precise;
- Also, the overall classification result is acceptable.

Therefore, a method of choosing the cuts is proposed.

## 6. The Cuts Selection Method Based on Histogram Segmentation

A comparison of the discretization results and thresholds obtained by the histogram segmentation produces the Cuts Selection Method. The MD algorithm is chosen because it produces the cuts set whose elements are close enough to the thresholds of histogram segmentation. Thresholds of the histogram segmentation are determined by the proposed Fixed Points Algorithm [6].

### 6.1. Fixed Points Algoritam

It is necessary to smooth the histogram. It is a result of the interpolation of new bars as the arithmetic mean of its neighbor bars with respect to their intensities. The procedure is correlated with basic geometry. A smooth modified multimodal distribution is obtained, having the same thresholds as the starting histogram.

An extraction of significant thresholds is proposed by defined segmentation criteria. The abscise of $g_{i}$ is a significant threshold; if

(1) $$\frac{g_{j}+g_{k}}{2}>\;K\;g_{i},$$

where $g_{i}$ is local minimum, $g_{j}$ and $g_{k}$ are the nearest local maximums, and K is a constant. The constant K is determined empirically, and optimal results are obtained by K = 1.2.

### 6.2. The Cuts Selection Method

**Figure 10:**
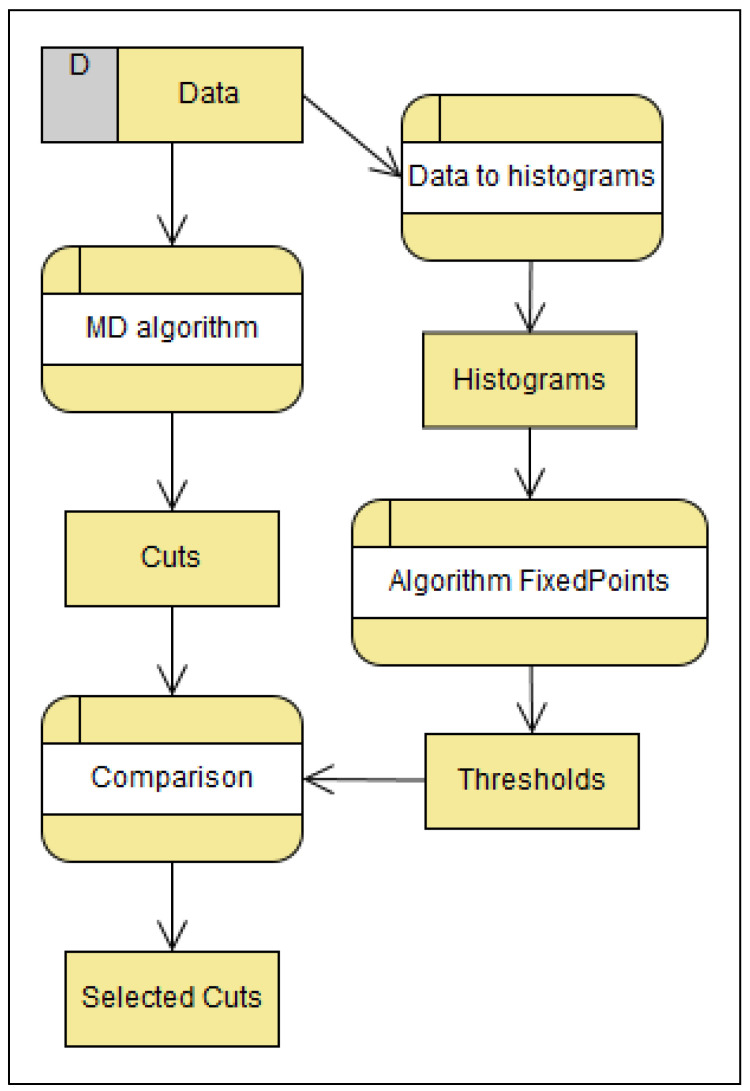
Cuts and thresholds comparison.

Initial data are processed by the MD Algorithm to produce the cuts. Simultaneously, initial data are transferred into histograms that are smoothed by the Fixed Points Algorithm, and significant thresholds are produced. A comparison of the cuts and the thresholds by the matching principle distinguishes the selected cuts. The Cuts Selection Method is shown in Figure 10. This method gives a smaller set of cuts, which is significant in order to preserve the consistency of the rules. 

The processes within the method are:

- Discretization by MD algorithm: Based on the properties of the MD algorithm in correlation with histogram segmentation, described in the Section 5, this algorithm is appropriate. Applying this algorithm to a data set results in a set of cuts.
- Data histograms: In order to obtain points that are compared with the cut points of the MD algorithm, it is necessary to make a histogram.
- Application of the Fixed Points Algorithm to histograms: From the histogram of the data, the Fixed Points Algorithm gives significant segmentation thresholds.
- Cuts and thresholds comparison: Of all the cuts obtained by the MD algorithm, those matching the significant thresholds of the multimodal distribution are selected.

## 7. Application of the Cuts Selection Method on the Particular Data Sets

The detailed application of classification methods will be presented in two parts. Initially, the cut segmentation method was performed on the Wireless Indoor Localization Data Set. Finally, classification results for 10 data sets are compared and shown for three methods: MD algorithm, Cuts Selection Method and Naïve Bayes algorithm.

### 7.1. Application of the Cuts Selection Method on the Wireless Indoor Localization Data Set

Based on the previous considerations and the Cuts Selection Method, a database with attributes from the second distribution type was examined. The obtained results were applied to select the optimal number of cuts. The Wireless Indoor Localization Data Set has seven condition attributes, a decision attribute with four possible values, a total of 2,000 instances, and five condition attributes that have a multimodal distribution (see Figure 8). Of all the cuts obtained by the MD algorithm, those matching the thresholds of the multimodal distribution are marked (see Table 4).

**Figure 11:**
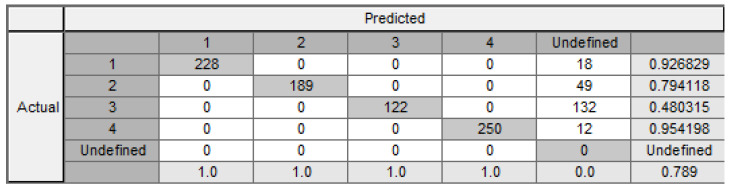
Confusion matrix with all cuts.

The classification result is calculated as described in Section 5.3, and the inconsistency is considered as the ratio of imprecise rules and all rules. The following conclusions are obtained:

The MD algorithm generated 384 rules, while the overall classification result was 78.9%. It should be noted that the consistency is 100%. Therefore, the percentage of the imprecise rules is 0%. The confusion matrix is shown in Figure 11.

**Figure 12:**
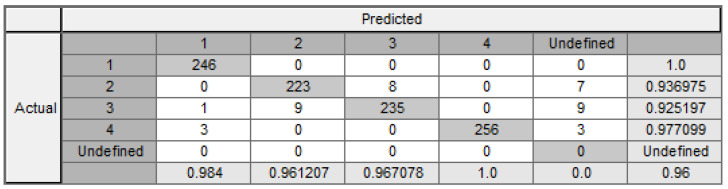
Confusion matrix with threshold cuts.

If segmentation thresholds marked in Table 4 are considered (nine cuts indicated in Figure 8), then only 72 rules are generated, and the overall classification result is 96% with 97.5% of consistency, which is an excellent result. Therefore, the percentage of the imprecise rules is 2.5%. The confusion matrix is shown in Figure 12.

The marked cuts in Table 4 represent the most significant of the segmentation thresholds of the multimodal distribution. If some additional segmentation thresholds are considered (thresholds that are not included in Table 4), then the result of the classification will also be greater than 78.9%, and the consistency will grow with the choice of a larger number of thresholds.

### 7.2. Comparation of the Classification Results Obtained by the Three Algorithms

All 10 considered data sets are classified by the following three algorithms/methods (see Table 5):

- The MD algorithm within the RST;
- The new Cuts Selection Method within the RST, based on the MD algorithm;
- The Naïve Bayes algorithm based on probability.

The Naïve Bayes algorithm is selected as the most common tool for data classification. Such an extensive comparison is very valuable. Datasets are divided in two sets, the training set and the test set (50%:50%).

The method was applied only for the bases having cut points for reduction within the attribute with multimodal distributions. The Cuts Selection Method achieves improved classification results.

## 8. Discussion and Conclusions

The importance of the thresholds of data histograms in data discretization is confirmed. The MD algorithm is “compatible” with the indiscernibility relation built into the RST. It uses a discernibility matrix to generate cut points. Therefore, it was expected that this algorithm would give cut points that have a better final score in RST classification. The entropy algorithm is great if used to fully generate decision trees. In this paper, it was experimentally shown that the entropy algorithm is not suitable for data preprocessing within RST as an MD algorithm. Therefore, the MD algorithm is more suitable for adjustment.

The general idea of segmentation data histogram has been employed in data preprocessing with the aim of discretization. Experiments within this work prove that discretization produced by the Cut Selection Method distinguishes the most significant cuts that preserve the consistency of the rules.

If the data are discretized by the MD algorithm, the obtained rules are slightly more consistent, and hence more precise compared with the Cut Selection Method. At the same time, the classification results are significantly better in the case of the Cut Selection Method, which is of greater interest.

The classification produced by the use of the Cut Selection Method is shifted towards the effects of the well-known Naïve Bayes algorithm.

Selecting cuts that match the thresholds of histogram segmentation allows for effective discretization. The indiscernibility relation on which the MD algorithm is based is clearly visible in the histogram segmentation: the obtained results emphasize the existence of a dependency between the type of histogram, the reduct, and the consistency of the obtained rules.

Further investigation will be pointed towards the creation of a discretization algorithm in accordance with histogram segmentation.

## Figures and Tables

**Table 1:** Membership to a specific histogram type.

Data Bases	No. of Numeric Condition Attributes	No. of Object	No. of I Type Attributes	No. of II Type Attributes
Iris	4	150	2	2
Blood Trans. Ser. Cen.	4	748	2	2
Banknote Authent.	4	1372	0	4
Glass Identification	9	214	4	5
Wilt Data Set	5	4339	5	0
Breast Cancer Wiscon.	9	699	1	8
Cardiotocography	22	2126	17	5
Statlog (Austr. Cred.)	6	690	4	2
Haberman’s Survival	3	306	2	1
Wireless Indoor Local.	7	2000	2	5

**Table 2:** Number of attributes and number of reduct attributes by type of histograms.

Data Sets	No. of Attr. I Type	No. of Attr. II Type	Entropy—No. of Reduct Attr. I Type	Entropy—No. of Reduct Attr. II Type	MD—No. of Reduct Attr. I Type	MD—No. of Reduct Attr. II Type
Iris	1	3	1	1	0	2
Blood Trans. Ser. Cen.	2	2	1	2	1	2
Banknote Authent.	0	4	0	3	0	3
Glass Identification	4	5	1	4	3	5
Wilt Data Set	5	0	1	0	4	0
Breast Cancer Wiscon.	1	8	1	7	1	5
Cardiotocography	17	5	5	5	0	3
Statlog (Austr. Cred.)	4	2	3	2	2	2
Haberman’s Survival	2	1	1	1	2	1
Wireless Indoor Local.	2	5	2	3	2	5

**Table 3:** Classification results.

Data Bases		MD Algorithm	Entropy
Iris	Number of rules	6	26
	Imprecise rules percent	0%	0%
	Confusion matrix	0.96	0.67
Blood Trans. Ser. Cen.	Number of rules	266	32
	Imprecise rules percent	4%	31%
	Confusion matrix	0.34	0.74
Banknote Authent.	Number of rules	67	432
	Imprecise rules percent	0%	0%
	Confusion matrix	0.97	0.45
Glass Identification	Number of rules	88	96
	Imprecise rules percent	0%	2%
	Confusion matrix	0.34	0.24
Wilt Data Set	Number of rules	215	113
	Imprecise rules percent	0%	0%
	Confusion matrix	0.96	0.98
Breast Cancer Wiscon.	Number of rules	129	103
	Imprecise rules percent	0%	5%
	Confusion matrix	0.77	0.70
Cardiotocography	Number of rules	811	811
	Imprecise rules percent	0%	0%
	Confusion matrix	0.39	0.33
Statlog (Austr. Cred.)	Number of rules	335	245
	Imprecise rules percent	0%	4%
	Confusion matrix	0.08	0.39
Haberman’s Survival	Number of rules	141	9
	Imprecise rules percent	0%	33%
	Confusion matrix	0.15	0.75
Wireless Indoor Local.	Number of rules	384	530
	Imprecise rules percent	0%	0%
	Confusion matrix	0.79	0.57

**Table 4:** Cuts obtained by the MD algorithm.

Attribute	Cut
1	**−54.5**
1	−48.5
1	**−44.5**
2	−58.5
2	−57.5
2	−54.5
2	−52.5
3	−54.5
4	**−56.5**
4	−48.5
4	**−46.5**
5	**−69.5**
5	−63.5
5	−62.5
5	**−56.5**
6	**−81.5**
7	**−83.5**
7	**−78.5**

**Table 5:** Classification results.

Data Bases	RST (MD Algorithm)	RST (Cuts Selection Method)	Naïve Bayes Algorithm
Iris	96%	96%	99%
Blood Trans. Ser. Cen.	34%	71%	76%
Banknote Authent.	97%	98%	86%
Glass Identification	34%	41%	35%
Wilt Data Set	96%	96%	98%
Breast Cancer Wiscon.	77%	-	97%
Cardiotocography	39%	-	88%
Statlog (Austr. Cred.)	8%	15%	81%
Haberman’s Survival	15%	28%	73%
Wireless Indoor Local.	79%	96%	98%

## References

[1] Shearer C. (2000). The CRISP-DM model: The new blueprint for data mining. J. Data Warehous..

[2] Ismail M.K., Ciesielski V. An Empirical Investigation of the Impact of. Discretization on Common Data Distributions. In Proceedings of the Third International Conference on Hybrid Intelligent Systems (HIS’03): Design and Application of Hybrid Intelligent Systems.

[3] Nguyen H.S. (2006). Approximate boolean reasoning: Foundations and applications in data mining. Transactions on Rough Sets V.

[4] Gama J., Pinto C. Discretization from Data Streams: Applications to Histograms and Data Mining. Proceedings of the 2006 ACM Symposium on Applied computing.

[5] Chang J.H., Fan K.C., Chang Y.L. (2002). Multi-modal gray-level histogram modeling and decomposition. Image Vis. Comput..

[6] Ognjenovic V. (2016). Approximative Discretization of Table-Organized Data. Ph.D. Thesis.

[7] Tan K.S., Ashidi M.I.N. (2001). Color image segmentation using histogram thresholding—Fuzzy C-means hybrid approach. Pattern Recognit..

[8] Gonzalez R.C., Woods R.E. (2002). Digital Image Processing.

[9] Sahoo P.K., Soltani S. (1988). A survey of thresholding techniques. Comput. Vis. Graph. Image Process..

[10] Kwon S.H. (2004). Threshold selection based on cluster analysis. Pattern Recognit. Lett..

[11] Gopalakrishnan S., Kandaswamy A. (2018). Automatic Delineation of Lung Parenchyma Based on Multilevel Thresholding and Gaussian Mixture Modelling. Comput. Model. Eng. Sci..

[12] Arifin Z., Asano A. (2006). Image segmentation by histogram thresholding using hierarchical cluster analysis. Pattern Recognit. Lett..

[13] Mohapatra S., Patra D., Kumar K. Blood microscopic image segmentation using rough sets. Processing of the 2011 International Conference on Image Information Processing (ICIIP).

[14] Xie C.H., Liu Y.-J., Chang J.-Y. (2015). Medical image segmentation using rough set and local polynomial regression. Multimed. Tools Appl..

[15] Hafemann L.G., Sabourin R., Oliveira L.S. (2017). Learning features for offline handwritten signature verification using deep convolutional neural networks. Pattern Recognit..

[16] Rosin P.L. (2001). Unimodal thresholding. Pattern Recognit..

[17] UCI (2015). UC Irvine Machine Learning Repository. https://archive.ics.uci.edu/ml/index.html.

[18] EasyFit Software (2015). Product Specification. http://www.mathwave.com/products/easyfit_desc.html.

[19] Fayyad U.M., Irani K.B. The Attribute Selection Problem in Decision Tree Generation. Proceedings of the 13th International Joint Conference on Artificial Intelligence.

[20] Øhrn A., Komorowski J., Skowron A., Synak P., Polkowski L., Skowron A. (1998). The ROSETTA, software system. Rough Sets in Knowledge Discovery 2. Applications, Case Studies and Software Systems, Number 19 in Studies in Fuzziness and Soft Computing.

[21] Shannon C.E. (1948). A Mathematical Theory of Communication. Bell Syst. Tech. J..

[22] Dobrilovic D., Ognjenovic V., Berkovic I., Radosav D. Analyses of WSN/UAV network configuration influences on 2.4 GHz IEEE 802.15.4 signal strength. Proceedings of the 2021 International Telecommunications Conference (ITC-Egypt).

[23] Lover R. (2008). Elementary Logic: For Software Development.

